# Field evaluation of a sing‐dose bivalent vaccine of porcine circovirus type 2b and *Mycoplasma hyopneumoniae*

**DOI:** 10.1002/vms3.420

**Published:** 2021-01-02

**Authors:** Siyeon Yang, Yongjun Ahn, Taehwan Oh, Hyejean Cho, Kee Hwan Park, Chanhee Chae

**Affiliations:** ^1^ College of Veterinary Medicine Department of Veterinary Pathology Seoul National University Seoul Republic of Korea

**Keywords:** enzootic pneumonia, *Mycoplasma hyopneumoniae*, porcine circovirus type 2, porcine circovirus‐associated disease, subclinical porcine circovirus type 2 infection

## Abstract

**Background:**

The field efficacy of a bivalent vaccine containing porcine circovirus type 2b (PCV2b) and *Mycoplasma hyopneumoniae* was evaluated on three pig farms.

**Methods:**

Three pig farms were used, two of which had a history of subclinical PCV2 and clinical *M. hyopneumoniae* infections between 84 and 126 days of age while concurrent porcine circovirus‐associated disease and clinical *M. hyopneumoniae* infection between 70 and 105 days of age. Each farm vaccinated pigs with a single dose of a bivalent vaccine at 10 days of age while unvaccinated pigs were administered a single dose of phosphate buffered‐saline at the same age.

**Results:**

Vaccination improved growth performance and reduced clinical scores significantly (*p* < .05) when compared with unvaccinated animals. The amount of PCV2d loads in blood and *M. hyopneumoniae* loads in nasal swabs of vaccinated animals were also significantly lower (*p* < .05) when compared with unvaccinated animals. Immunologically, vaccinated groups elicited a significantly higher (*p* < .05) level of protective immunity against PCV2d such as neutralizing antibodies and interferon‐γ secreting cells (IFN‐γ‐SC), as well as protective immunity against *M. hyopneumoniae* such as IFN‐γ‐SC when compared with unvaccinated animals. Pathologically, vaccination significantly lowered (*p* < .05) the scores of *M. hyopneumoniae*‐induced pneumonia and PCV2‐associated lymphoid lesions when compared with unvaccinated animals.

**Conclusions:**

The evaluated bivalent vaccine provided good protection against PCV2d and *M. hyopneumoniae* infection under field conditions.

## Graphical abstract

Bivalent vaccine was effective in improving growth performance and protecting pigs against PCV2d and *M. hyopneumoniae* infection under field conditions.

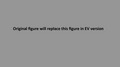

## 1 INTRODUCTION

Porcine circovirus type 2 (PCV2) and *Mycoplasma hyopneumoniae* are two worldwide economically important dominating pathogens. PCV2 is a very small DNA virus which can be divided into at least eight different genotypes (‘a’ to ‘h’) based on its sequence identity in open reading frame 2 (ORF2) (Bao et al., 2018; Franzo & Segales, 2018; Wang et al., 2009; Xiao et al., 2015). Currently, PCV2d is the predominant genotype in Asia and North America (Franzo & Segales, 2018). PCV2 causes different clinical diseases and syndromes which are collectively referred to as porcine circovirus‐associated disease (PCVAD). In Korea, PCV2 infection is so widespread that essentially all pig herds are infected with the pathogen. The decrease in PCVAD outbreaks since 2008 is attributed to the successful introduction of efficacious PCV2 vaccines to the market. Near or fully asymptomatic subclinical PCV2 infection remains the most common disease, leading to poor growth that increases days to market as described by other countries (Alarcon et al., 2013; Alarcon et al., 2013; Kurmann et al., 2011; Segalés, 2012).

Mycoplasmas including *Mycoplasma hyopneumoniae* are the smallest self‐replicating bacteria and are distinguished by the lack of a cell wall that is present in most bacteria (Razin et al., 1998). *M*. *hyopneumoniae* is the primary causative agent for enzootic pneumonia; one of the most widespread and costly diseases in the swine respiratory disease worldwide today. It is characterized by significant economic losses due to slower growth and poor feed conversion.

Coinfection with PCV2 and *M. hyopneumoniae* is one of the most devastating and damaging combinations of pig diseases. Vaccination against PCV2 and *M. hyopneumoniae* is one of the most cost‐effective strategies and is widely used in Asian pork production. A new bivalent vaccine containing PCV2b and *M. hyopneumoniae* (Circo/MycoGard, Pharmgate Animal Health, Wilmington, NC, USA) was first introduced into the Asian market. In particular, this bivalent vaccine is clinically interesting because it contains the PCV2b genotype. Genetically, PCV2b and PCV2d (initially called a mutant of PCV2b) are more closely related than PCV2a and PCV2d (Xiao et al., 2015). The objective of this study was to evaluate the new single‐dose bivalent vaccine containing PCV2b and *M. hyopneumoniae* based on clinical, microbiological, immunological and pathological analysis under field conditions in accordance with the registration guidelines of the Republic of Korea's Animal, Plant & Fisheries Quarantine & Inspection Agency (QIA, http://www.qia.go.kr).

## 2 MATERIALS AND METHODS

### 2.1 Farm history

The clinical field trial was conducted on three farms (A, B and C) between May and October of 2019. Farms A, B and C were each a 380‐sow, 160‐sow and 430‐sow (respectively), farrow‐to‐finish swine farm with an all‐in‐all‐out production system. All three farms are located in Chungcheongnam‐do. Sows from these selected farms had not received vaccines against PCV2 or *M. hyopneumoniae*, while all piglets from this production system were immunized against both pathogens. Farms A and C were selected based on history of concurrent subclinical PCV2 infection and enzootic pneumonia. Herds approximately 12–18 weeks of age displayed subclinical PCV2 infection and enzootic pneumonia characterized by respiratory signs and growth retardation which were mainly associated with a marked increase in the mortality rate (approximately 7% and 5% of Farms A and C, respectively) from 21 to 140 days of age. Lungs were examined at the slaughterhouse where it was confirmed that 12 of 20 Farm A pigs and 15 of 20 Farm C pigs had mycoplasmal pneumonia lesions based on scoring from a previous method (Goodwin & Whittlestone, 1973). Submitted cases from two farms met the definition of subclinical PCV2 infection (Segalés, 2012) including decreased average daily gain without overt clinical signs, no or minimal histopathological lesions in inguinal lymph nodes and the presence of low amounts of PCV2 in inguinal lymph nodes by immunohistochemistry in 4 of 5 suspected pigs from Farm A and 5 out of 5 suspected animals on Farm C. Histopathological mycoplasmal lung lesions were observed in three of the five pigs submitted from Farm A, and four of the five pigs submitted from Farm C. Farm B was selected based on a history of concurrent clinical PCVAD and enzootic pneumonia. Farm C swine displayed clinical signs of PCVAD and *M. hyopneumoniae* infection characterized by wasting, respiratory signs and growth retardation which were mainly associated with a marked increase in the mortality rate (approximately 13%) at approximately 13–18 weeks of age. A lung examination was performed at the slaughterhouse, which confirmed that 8/10 pigs had mycoplasmal pneumonia lesions. Submitted cases from Farm B met the definition of PCVAD (Chae, 2005) including wasting and growth retardation, lymphoid granulomatous inflammation with grape‐like intracytoplasmic inclusion bodies and the presence of PCV2 antigen in lymphoid lesions by immunohistochemistry in 4 of 5 suspected animals.

### 2.2 Clinical field study design

The experimental design for the field study strictly adhered to the guidelines set by QIA. QIA guidelines required that 20 piglets (male = 10 and female = 10) were selected and assigned to each group of vaccinated and unvaccinated animals. In an effort to minimize sow variation, four to six 7‐day‐old piglets were randomly selected from each sow and assigned evenly to either the vaccinated or unvaccinated group using the random number generation function in Excel (Microsoft Corporation, Redmond, WA, USA). The pigs in the vaccinated (VacA, VacB and VacC) groups were injected intramuscularly in the right side of the neck with 1.0 ml of the bivalent vaccine containing PCV2b and *M. hyopneumoniae* (Circo/MycoGard, Serial No: CMG‐18007, Expiration date: 02.28.2020, Pharmgate Animal Health) at 10 days of age. An equal volume of phosphate‐buffered saline (PBS, 0.01M, pH 7.4) was injected in the same anatomical location in pigs of the unvaccinated (UnVacA, UnVacB and UnVacC) groups. Both vaccinated and unvaccinated pigs were comingled before they were randomly distributed into four different pens kept within one room. Each pen contained 10 pigs with a similar proportion of each treatment per pen. Pens were identical in design and equipment which included free access to a feed and water trough. Standard farm procedures were followed regarding the feeding and watering of study animals. Blood and nasal swabs were collected at 0 (10 days of age), 18 (28 days of age), 39 (49 days of age), 81 (91 days of age) and 102 (112 days of age) days post‐vaccination (dpv). All methods used in this study were approved by the Seoul National University Institutional Animal Care and Use Committee.

### 2.3 Clinical observations

Pigs were monitored and scored weekly for clinical signs as previously described (Seo et al., 2012). Briefly, scoring was defined as follows 0 (normal), 1 (rough haircoat), 2 (rough haircoat and dyspnoea), 4 (severe dyspnoea and abdominal breathing) and 6 (death). Observers were blinded to vaccination status.

### 2.4 Growth performance

The live weight of each pig was measured at 10 (0 dpv), 70 (60 dpv), 112 (102 dpv) and 175 (165 dpv) days of age. The average daily weight gain (ADWG; gram/pig/day) was analysed over three time periods: (1) between 10 and 70 days of age, (2) between 70 and 112 days of age and (3) between 112 and 175 days of age. ADWG during the different production stages was calculated as the difference between the starting and final weight divided by the duration of the stage. Data for dead or removed pigs were included in the calculation.

### 2.5 Quantification of PCV2d DNA in blood

DNA was extracted from serum samples by use of a commercial kit (QIAamp DNA Mini Kit, QIAGEN, Valencia, CA, USA). Real‐time PCR was used to quantify the number of genomic DNA copies for PCV2d (Jeong et al., 2015).

### 2.6 Quantification of *M. hyopneumoniae* in nasal swabs

DNA was extracted from nasal swabs by use of a commercial kit (QIAamp DNA Mini Kit, QIAGEN). Real‐time PCR was used to quantify the number of genomic DNA copies for *M. hyopneumoniae* was quantified by real‐time PCR (Dubosson et al., 2004).

### 2.7 Serology

Enzyme‐linked immunosorbent assay (ELISA) was used to test for both PCV2 and *M. hyopneumoniae* antibodies with commercial ELISA kits (SERELISA PCV2 Ab Mono Blocking, Synbiotics, Lyon, France, and *M. hyo* Ab test, IDEXX Laboratories Inc. Westbrook, ME, USA). Serum samples were considered as positive for anti‐PCV2 antibodies if the reciprocal ELISA titre was > 350 and as positive for *M. hyopneumoniae* antibody if the sample‐to‐positive (S/P) ratio was ≥ 0.4 in accordance with the manufacturer's instructions for each kit. Serum samples were also tested for neutralizing antibodies (NA) against PCV2d (Pogranichnyy et al., 2000).

### 2.8 Enzyme‐linked immunospot assay

Enzyme‐linked immunospot assay was used to measure the numbers of *M. hyopneumoniae* and PCV2d‐specific interferon‐γ secreting cells (IFN‐γ‐SC). Peripheral blood mononuclear cells (PBMC) were stimulated using the aforementioned challenge *M. hyopneumoniae* and PCV2d strains (Jeong et al., 2018) with results reported as the numbers of IFN‐γ‐SC per million PBMC.

### 2.9 Pathology

The severity of macroscopic lung lesions was scored by two pathologists (Chae and one graduate student) at the Seoul National University (Seoul, Republic of Korea) to estimate the percentage of the lung affected by pneumonia. Scoring was performed out of 100 total possible points over the entire lung as follows: 10 points each to the right cranial lobe, right middle lobe, left cranial lobe and left middle lobe; 27.5 points each to the right caudal lobe and left caudal lobe and 5 points to the accessory lobe (Halbur et al., 1995).

Collected lung and lymphoid tissue sections were examined by two blinded veterinary pathologists (Chae and one graduate student). The severity of peribronchiolar and perivascular lymphoid tissue hyperplasia was assessed by scoring mycoplasmal pneumonia lesions (0 to 6) (Opriessnig et al., 2004). Mycoplasmal pneumonia lesions were confirmed by real‐time PCR from lung lesions (Dubosson et al., 2004). The severity of lymphoid lesions was scored (0 to 5) based on the severity of lymphoid depletion and granulomatous inflammation (Kim & Chae, 2004).

### 2.10 Immunohistochemistry

Immunohistochemistry for PCV2 was performed as previously described (Park et al., 2013). For the morphometric analyses of immunohistochemistry, three sections were cut from each of three blocks of tissue from lymph node of each pig. The slides were analysed using the NIH Image J 1.45s Program (http://imagej.nih.gov/ij/download.html) to obtain the quantitative data. For the analysis of PCV2, 10 fields were randomly selected, and the number of positive cells per unit area (0.95 mm^2^) was determined as previously described (Kim et al., 2003). The mean values were also calculated.

### 2.11 Statistical analysis

Prior to statistical analysis, real‐time PCR and neutralizing antibody data were transformed to log_10_ and log_2_ values respectively. Data were tested for the normal distribution using the Shapiro‐Wilk test and either the Student's *t* test or Mann‐Whitney test were used to examine whether significant statistical differences existed between the two groups at each time point. The student's *t* test was conducted to compare the difference between the two groups, only if the normality assumption was met, while the Mann‐Whitney test was performed to compare the differences between the two groups when the normality assumption was not met. A value of *p* < .05 was considered to be significant.

## 3 RESULTS

### 3.1 Clinical evaluation

**FIGURE 1:**
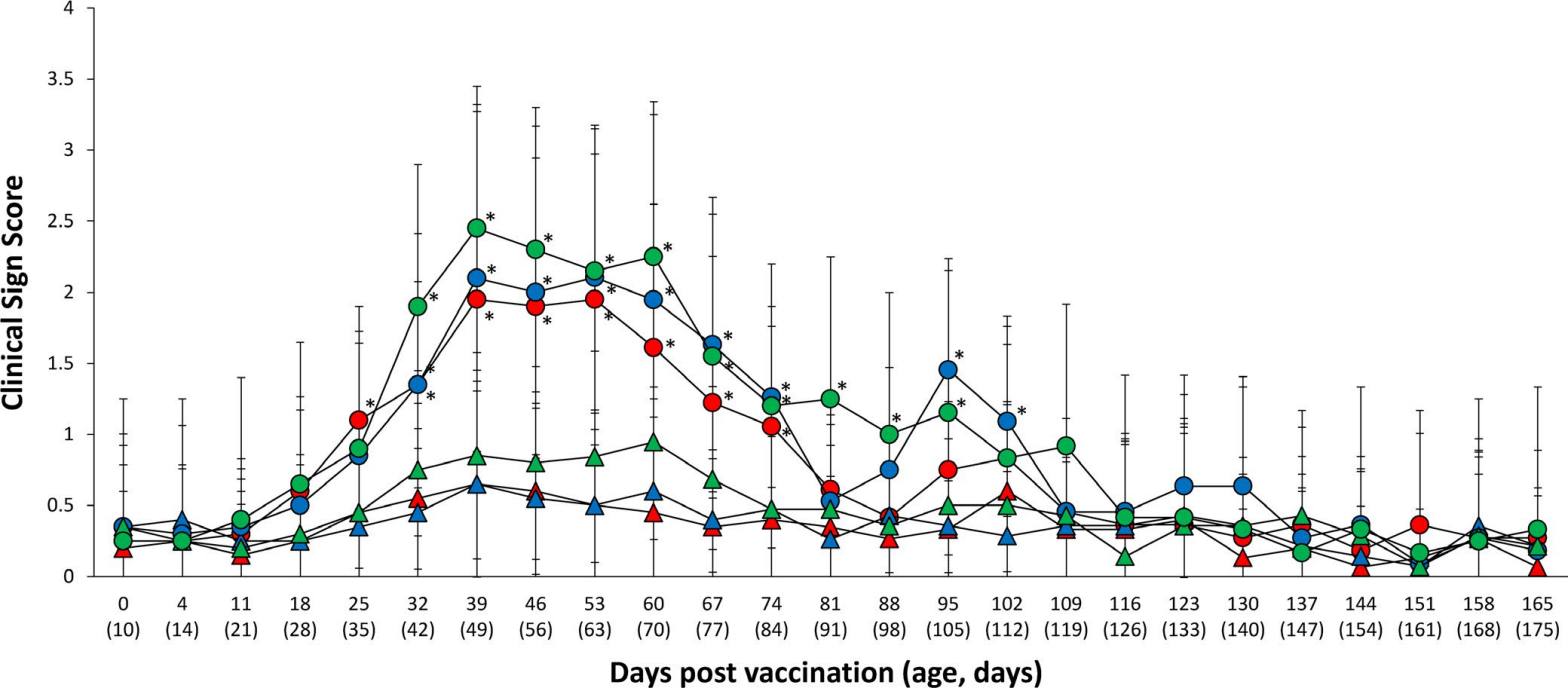
Mean respiratory score from VacA (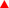) and UnVacA (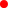), VacB (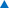) and UnVacB (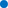), and VacC (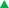) and UnVacC (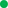) groups. Variation is expressed as the standard deviation. ^*^Significant difference (*p* < .05) between vaccinated and unvaccinated group within the same farm

The vaccinated group on Farm A had the significantly lower (*p* < .05) clinical scores between 25 and 74 dpv when compared with the unvaccinated group. Clinical scores were significantly lower (*p* < .05) in the Farm B vaccinated group between 32 and 74 dpv, and at 95 and 102 dpv when compared with the unvaccinated group. Farm C clinical scores were significantly lower (*p* < .05) between 32 and 95 dpv in the vaccinated group when compared with the unvaccinated group (Figure 1).

### 3.2 Growth performance

A significant difference (*p* < .05) in the ADWG was observed on Farm A between vaccinated and unvaccinated group between 10 and 70, between 70 and 112 and between 112 and 175 days of age. The ADWG in the Farm B vaccinated group during the 10–70 and 112–175 days period was significantly higher (*p* < .05) than that of the unvaccinated group. The overall growth performance of all three farms (10 to 175 days of age) of vaccinated groups was significantly higher (*p* < .05) than that of unvaccinated group (Table 1).

**TABLE 1:** Average daily weight gain (ADWG), mortality rate and pathology between vaccinated (Vac) and unvaccinated (UnVac) animals on three Farms

	Age (day)	Farm A		Farm B		Farm C	
		VacA	UnVacA	VacB	UnVacB	VacC	UnVacC
ADWG (gram/pig/day)	10–70	369 ± 20*	344 ± 22	375 ± 20*	350 ± 26	360 ± 27	343 ± 29
	70–112	751 ± 37*	720 ± 25	738 ± 36	733 ± 30	770 ± 43	761 ± 59
	112–175	774 ± 28*	748 ± 37	771 ± 28*	742 ± 36	741 ± 36	730 ± 31
	10–175	620 ± 10*	594 ± 14	620 ± 15*	600 ± 15	610 ± 27*	343 ± 29
Initial body weight (kg)	10	3.3 ± 0.3	3.4 ± 0.2	3.3 ± 0.4	3.4 ± 0.3	3.3 ± 0.4	3.3 ± 0.4
Market body weight (kg)	175	105.6 ± 1.6*	101.4 ± 2.4	105.5 ± 2.4*	102.1 ± 2.5	104.1 ± 1.1*	101.5 ± 2.4
Mortality rate		0/20	4/20	1/20	4/20	1/20	3/20
Lung lesion score							
Macroscopic	175	12 ± 4.06*	42 ± 10.95	15 ± 2.71*	48 ± 7.76	16 ± 5.24*	53 ± 14.60
Microscopic	175	0.8 ± 0.28*	2.5 ± 0.83	0.8 ± 0.33*	3.0 ± 0.50	1.3 ± 0.33*	2.7 ± 0.54
Lymphoid lesion score	175	0.4 ± 0.41*	1.6 ± 0.43	1.0 ± 0.48*	2.3 ± 0.44	0.9 ± 0.54*	1.7 ± 0.41
PCV2‐antigen positive cells	175	3 ± 1.08*	7.6 ± 6.14	4.1 ± 1.34*	7.6 ± 3.1	4.6 ± 2.13*	16 ± 4.40

* Significant difference (*p* < .05) between vaccinated and unvaccinated groups within the same farm.

### 3.3 Mortality

Farm A mortality included one unvaccinated pig that died of suppurative leptomeningitis as determined by the isolation of *Streptococcus suis* from the meninges at 63 days of age, and one unvaccinated pig that died of Glasser's disease as determined by the isolation of *Glaesseralla parasuis* from the pericardium at 70 days of age. Two additional unvaccinated pigs died of enzootic pneumonia as determined by a combination of *M. hyopneumoniae* that was detected with PCR and *Pasteurella multocida* that was isolated from the lungs at 92 and 117 days of age. Causes of death on Farm B included one vaccinated pig that died of hemorrhagic enteritis by unknown aetiology at 84 days of age, and one unvaccinated pig that died of colibacillosis that was determined by the isolation of *Escherichia coli* from the small intestine at 53 days of age. Two additional unvaccinated pigs died of suppurative bronchopneumonia and *M. hyopneumoniae* that was detected by PCR and *Trueperella pyogenes* that was isolated from the lungs at 89 and 90 days of age. A fourth unvaccinated pig died of pneumonia, as determined by *M. hyopneumoniae* detection with PCR. PCV2 was also detected by immunohistochemistry methodology in this fourth unvaccinated pig at 104 days of age. Farm C mortality included one vaccinated pig that died of exudative epidermitis that was determined by the isolation of *Staphylococcus hyicus* from skin at 62 days of age, and one unvaccinated pig that died of pneumonic pasterellosis that was determined by the isolation of *P. multocida* from the lung at 93 days of age. A second unvaccinated pig died of pneumonia complications. *M. hyopneumoniae* was detected by PCR along with PCV2 as detected with immunohistochemistry methodology at 101 days of age in the second unvaccinated pig. A third and final unvaccinated pig died of suppurative bronchopneumonia, where *M. hyopneumoniae* was determined as the causative agent through PCR. *Trueperella pyogenes* was also isolated from the lung of this third unvaccinated pig at 111 days of age.

### 3.4 Quantification of PCV2d DNA in blood

**FIGURE 2:**
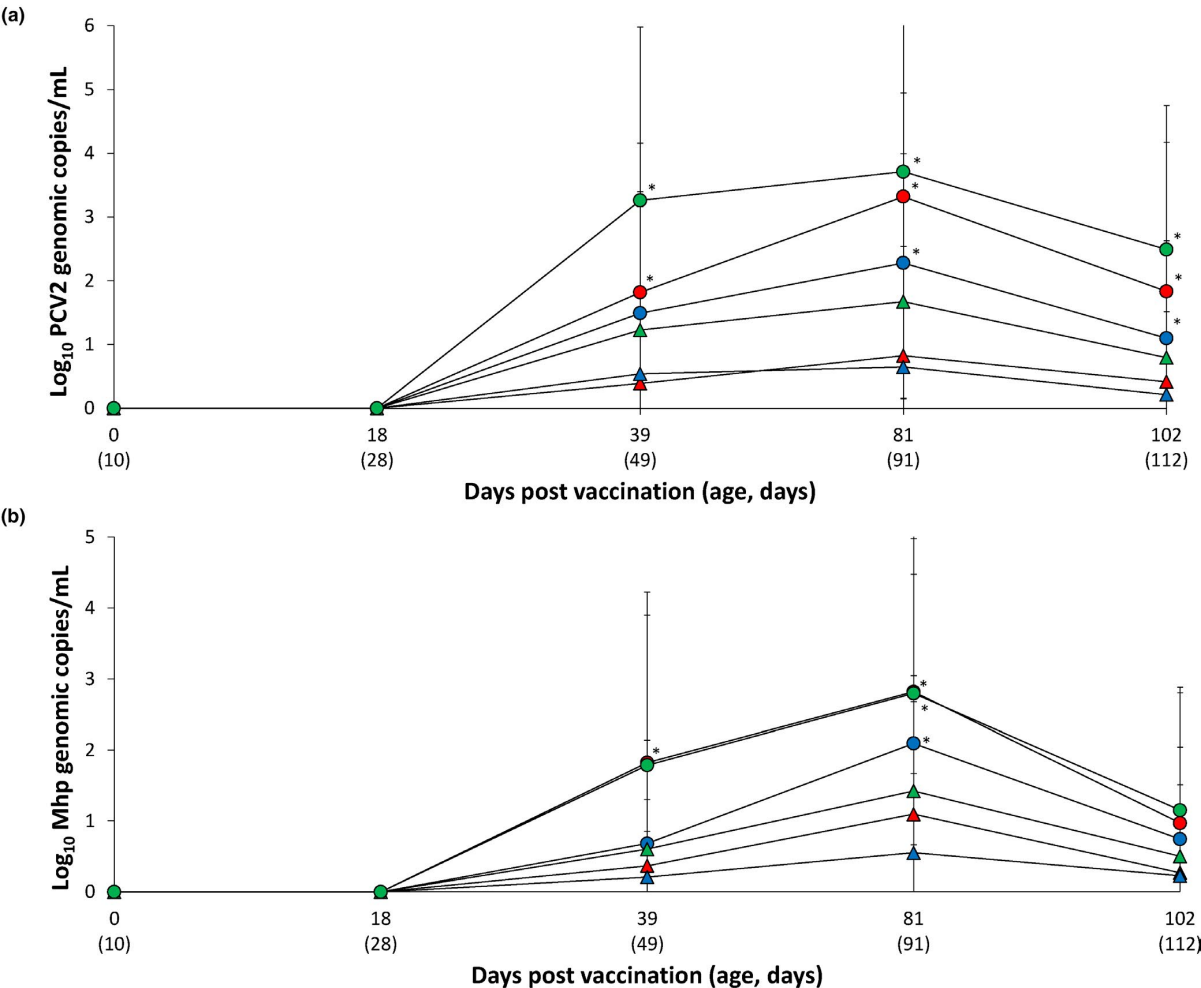
Mean values of the genomic copy number of PCV2d DNA in serum of pigs (a) and of *M. hyopneumoniae* DNA in nasal swab of pigs (b) from VacA (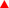) and UnVacA (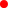), VacB (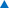) and UnVacB (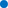), and VacC (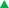) and UnVacC (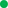) groups. Variation is expressed as the standard deviation. ^*^Significant difference (*p* < .05) between vaccinated and unvaccinated group within the same farm

PCV2 DNA was not detected in the blood of either vaccinated or unvaccinated animals at 0 and 18 dpv at any of the three farms. PCV2d was detected in blood at 39 dpv (49 days of age) at all three farms. Vaccinated pigs from Farms A and C had a significantly lower (*p* < .05) number of genomic copies of PCV2d in their blood at 39 dpv compared with that of unvaccinated animals. Vaccinated pigs from all three farms had a significantly lower (*p* < .05) number of genomic copies of PCV2d in their blood between 81 and 102 dpv compared with that of unvaccinated animals (Figure 2a).

### 3.5 Quantification of *M. hyopneumoniae* DNA in nasal swabs

*M. hyopneumoniae* DNA was not detected in nasal swabs from either vaccinated or unvaccinated animals at 0 and 18 dpv from any of the three farms. Vaccinated animals had a significantly lower (*p* < .05) number of *M. hyopneumoniae* genomic copies in their nasal swabs when compared with unvaccinated animals at 39 and 81 dpv (Farm A), and 81 dpv (Farms B and C) (Figure 2b).

### 3.6 Immunological responses against PCV2

**FIGURE 3:**
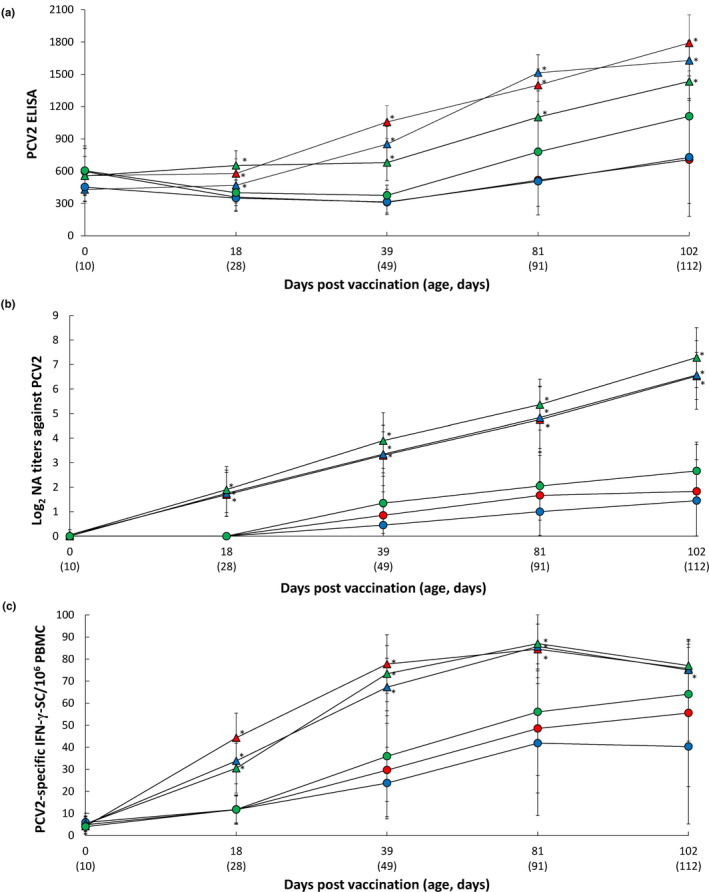
Mean values of the PCV2 ELISA titres (a), the PCV2d‐specific neutralizing antibodies (b) and PCV2d‐specific IFN‐γ‐SC/10^6^ PBMC (c) from VacA (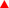) and UnVacA (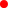), VacB (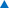) and UnVacB (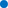), and VacC (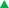) and UnVacC (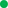) groups. Variation is expressed as the standard deviation. ^*^Significant difference (*p* < .05) between vaccinated and unvaccinated group within the same farm

At the time of vaccination (10 days of age; 0 dpv), significant differences between vaccinates and non‐vaccinates were not detected at any of the three farms in regard to anti‐PCV2 antibodies. The PCV2 ELISA titres (Figure 3a) and PCV2‐specific NA (Figure 3b) were significantly higher (*p* < .05) in the vaccinated group when compared with the unvaccinated group at 18–102 dpv in three farms. The mean frequencies of PCV2‐specific IFN‐γ‐SC remained at basal levels (< 20 cells/10^6^ PBMC) in both groups until 0 dpv. Thereafter, the mean number of PCV2‐specific IFN‐γ‐SC was significantly higher (*p* < .05) in the vaccinated group when compared with the unvaccinated group from 18 to 81 dpv (Farms A and C) and from 18 to 102 dpv at Farm B (Figure 3c).

### 3.7 Immunological responses against *M. hyopneumoniae*

**FIGURE 4:**
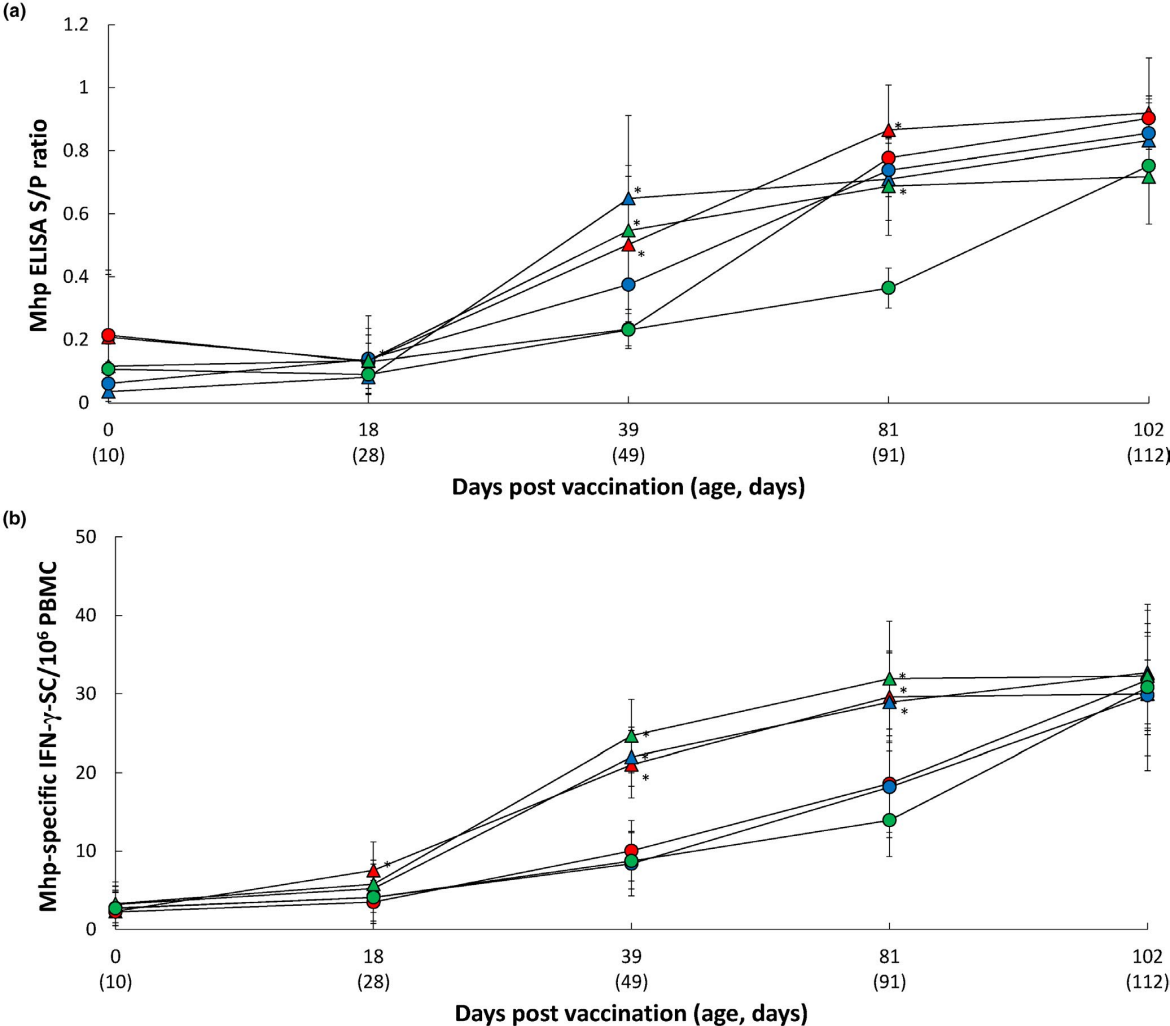
Mean values of the *M. hyopneumoniae* ELISA S/P ratio (a) and *M. hyopneumoniae*‐specific IFN‐γ‐SC/10^6^ PBMC (b) from VacA (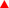) and UnVacA (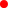), VacB (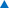) and UnVacB (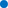), and VacC (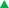) and UnVacC (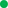) groups. Variation is expressed as the standard deviation. ^*^Significant difference (*p* < .05) between vaccinated and unvaccinated group within the same farm

At the time of vaccination (3 weeks of age; 0 dpv), anti‐*M. hyopneumoniae* antibodies were not detected in any of the groups or farms. Vaccinated animals from Farms A and C had significantly higher (*p* < .05) *M. hyopneumoniae* ELISA S/P ratios at 39 and 81 dpv (respectively) when compared with unvaccinated animals. Farm B vaccinated animals had a significantly higher (*p* < .05) *M. hyopneumoniae* ELISA S/P ratios at 18 and 39 dpv when compared with unvaccinated animals. The mean number of *M. hyopneumoniae*‐specific IFN‐γ‐SC remained at basal levels (< 20 cells/10^6^ PBMC) in both groups until 18 dpv (Figure 4a). The mean number of *M. hyopneumoniae*‐specific IFN‐γ‐SC was significantly higher (*p* < .05) in the vaccinated groups when compared with the unvaccinated groups from 18 to 81 dpv (Farm A) and from 39 to 81 dpv (Farms B and C) (Figure 4b).

### 3.8 Pathology

The results of lung and lymphoid lesion score are summarized in Table 1. Vaccinated pigs from all three farms had a significantly lower severity (*p* < .05) of *M. hyopneumoniae*‐induced pneumonia and PCV2‐associated lymphoid lesions when compared with unvaccinated pigs. Vaccinated pigs on three farms also had significantly lower numbers of PCV2 antigen‐positive cells within lymphoid lesions when compared with unvaccinated pigs.

## 4 DISCUSSION

In this field trial, vaccination against PCV2b and *M. hyopneumoniae* resulted in a statistically significant improvement in growth performance when compared with unvaccinated pigs in all three farms, regardless of whether the farm was experiencing a subclinical PCV2 and clinical *M. hyopneumoniae* concurrent infection (Farms A and C), or concurrent PCVAD and clinical *M. hyopneumoniae* infection (Farm B). Although Farm B received a PCV2a vaccine, PCVAD was not eradicated. Commercial PCV2a‐based vaccines have proven to provide cross‐protection against PCV2d (Opriessnig et al., 2014, 2017; Park et al., 2019; Xiao et al., 2015), yet vaccine failure (contradicting this cross‐protection) due to PCV2d infection has also been reported in PCV2a‐vaccinated herds (Opriessnig et al., 2013; Ramos et al., 2015; Seo, Park, et al., 2014). Therefore, vaccines containing PCV2b may provide better protection against PCV2d, which is genetically closed to PCV2b. On the other hands, despite vaccination with *M. hyopneumoniae* on the three farms, clinical signs and mycoplasmal lung lesions were still observed in pigs at the time of slaughter. Similarly, bivalent‐vaccinated pig groups from all three farms exhibited some degree of clinical signs and mycoplasmal lung lesions at the time of slaughter. These results indicate that vaccination alone is not sufficient in protecting pigs from *M. hyopneumoniae*.

The most common age of piglet PCV2 vaccination occurs either at weaning or immediately after weaning (around 3–4 weeks of age). In contrast, the evaluated bivalent vaccine containing PCV2b and *M. hyopneumoniae* recommended administration to the piglets at 10 days old. This younger‐aged piglet was still immunologically mature enough to elicit active immunization after vaccination, as the onset of active immunity has been proven to begin as early as in 5‐day‐old piglets, post‐vaccination (O'Neill et al., 2011). Vaccination at such a young age may result in additional complications, however, as the piglets face potential interference of maternally derived antibodies (MDA) present at the time of vaccination. The role of PCV2 MDA plays in active immunity of piglets after vaccination is a somewhat controversial issue. It has been stated that PCV2 MDA can affect the age of PCV2 infection (Rose et al., 2012) while contradictory evidence exists that demonstrated that high PCV2 MDA titres (≥ 10 log_2_) could interfere with piglets’ active seroconversion after vaccination (Fort at al., 2009). In the present study, piglets with high ELISA (> 9 log_2_) or NA (> 7 log_2_) titres seemed to show interference with the development of the humoral immune response after vaccination. Most of the piglets (> 80% in three Farms) had lower ELISA (< 9 log_2_) or NA (< 7 log_2_) titres at the time of vaccination (data not shown). The data presented in this study support that the bivalent vaccine can elicit PCV2‐specific NA and IFN‐γ‐SC even in the presence of MDA. It is therefore concluded that a negative MDA effect on active immunization after vaccination could not have impacted the efficacy of the bivalent vaccine.

Optimal timing of vaccination against *M. hyopneumoniae* also continues to be debated because of the interference of MDA. *M. hyopneumoniae* may infect pigs within the first 3 weeks of life (Fano et al., 2007; Sibila et al., 2007). Similarly, *M. hyopneumoniae* is frequently detected in laryngeal swabs from suckling piglets in Korea (personal observation Dr. Chae). Therefore, an earlier vaccination against *M. hyopneumoniae* at less than 21 days old may be necessary in order to vaccinate piglets to prevent the onset of a natural infection. A number of studies have been conducted to evaluate early piglet vaccination. One experimental challenge study proved that vaccination of pigs at 7 days old with *M. hyopneumoniae* was effective in reducing lung lesions even in the presence of MDA at a titre considerably higher than what is typically seen in the field (Wilson et al., 2012, 2013). Moreover, protective immunity against *M. hyopneumoniae* is primarily cell mediated (Djordjevic et al., 1997; Thacker et al., 2000) and the present field trial proved that the bivalent vaccine elicited *M. hyopneumoniae*‐specific IFN‐γ‐SC even in the presence of MDA. Therefore, a MDA effect on active immunization after vaccination is less likely to have an impact on the efficacy of the bivalent vaccine.

The bivalent vaccine is able to elicit protective immunity against PCV2 and *M. hyopneumoniae*. PCV2‐specific NA and IFN‐γ‐SC are key needed component to reduce PCV2 viremia and lymphoid lesions (Chae, 2012; Fort at al., 2009; Seo et al., 2014). PCV2 viremia and lymphoid lesions results were significantly lower in the vaccinated groups when compared with controls in all three trial farms. Similarly, *M. hyopneumoniae*‐specific IFN‐γ‐SC plays a crucial role to reduce nasal shedding of *M. hyopneumoniae* and mycoplasmal lung lesions (Jeong et al., 2018; Park et al., 2016). In the presented field trials, vaccination resulted in the reduction in nasal shedding of *M. hyopneumoniae* and mycoplasmal lung lesions when compared with controls.

Vaccination against PCV2 and *M. hyopneumoniae* has becomes standard practice, and almost 100% and 80% of the Korean herds are vaccinated against PCV2 and *M. hyopneumoniae* respectively (Park et al., 2019). Use of a bivalent vaccine reduces the number of injections the pig receives, is more convenient than handling multiple products and increases administration efficiency. This bivalent vaccine containing PCV2b and *M. hyopneumoniae* provide good efficacy against PCV2d and *M. hyopneumoniae* on farms with concurrent subclinical PCV2 and clinical *M. hyopneumoniae* infection, and concurrent PCVAD and clinical *M. hyopneumoniae* infection.

## CONFLICT OF INTEREST

The authors declare no conflict of interests with respect to their authorship.

## AUTHOR CONTRIBUTION

Siyeon Yang: Conceptualization; Data curation; Investigation; Methodology. Yongjun Ahn: Conceptualization; Formal analysis; Investigation; Software. Taehwan Oh: Data curation; Resources; Software; Visualization. Hyejean Cho: Methodology; Software. Kee Hwan Park: Formal analysis; Validation. C. Chae: Conceptualization; Project administration; Supervision; Writing‐review & editing.

## Funding Information

The author's research was supported by Contract Research Funds (Grant no.550–20180050) of the Research Institute for Veterinary Science (RIVS) from the College of Veterinary Medicine and by the BK 21 FOUR Future Veterinary Medicine Leading Education and Research Center.

## ETHICAL STATEMENT

All of the methods were previously approved by the Seoul National University Institutional Animal Care and Use, and Ethics Committee. Sample collection was carried out according to the animal welfare code of Korea.

### Peer Review

The peer review history for this article is available at https://publons.com/publon/10.1002/vms3.420.

## Data Availability

The data that support the findings of this study are available from the corresponding author upon reasonable request.
